# Dermoscopy Observation of Bee Sting in a 3-Year-Old Girl

**DOI:** 10.4269/ajtmh.23-0188

**Published:** 2023-07-03

**Authors:** Nan Wang, Lin Ma, Ya Bin Zhou

**Affiliations:** Department of Dermatology, Beijing Children’s Hospital, Capital Medical University, National Center for Children’s Health, Beijing, China

**Figure 1.**
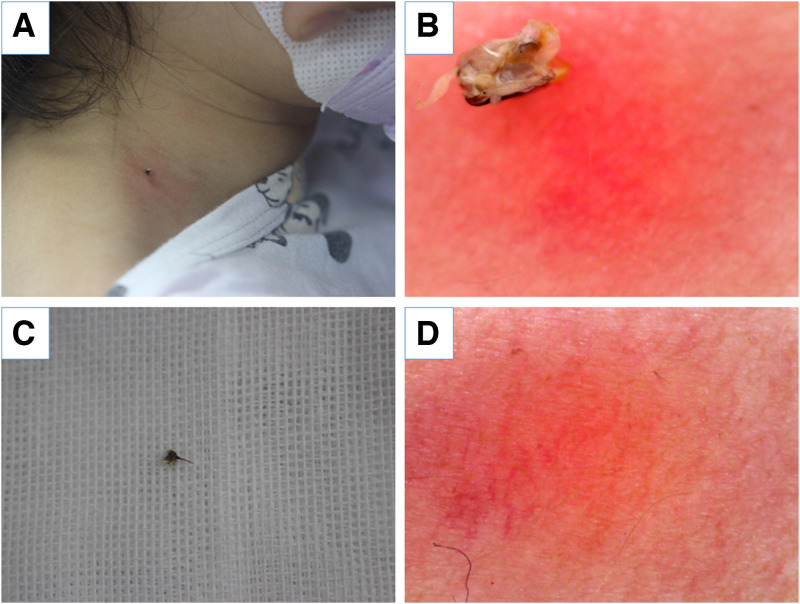
(**A**) A foreign body with surrounding edematous erythema was observed on the right side of the neck. (**B**) Honeybee body residue was observed by dermoscopy. (**C**) A stinger was removed. (**D**) An edematous erythema without residual stinger was observed by dermoscopy.

A 3-year-old girl presented to our outpatient clinic with a bee sting on her neck. One hour earlier, a honeybee had landed on her neck, and she had swatted it, resulting in a painful sting. The patient did not exhibit any symptoms of fever, cough, cold, nausea, vomiting, breathlessness, chest pain, palpitations, blackouts, or loss of consciousness, but she did report pain in the area where she was stung. On examination, we observed a foreign body on the right side of the patient’s neck (Figure 1A). Dermoscopy revealed that the foreign body was the residue of the honeybee’s body (Figure 1B). We removed the stinger by scraping it with a credit card (Figure 1C), and no residual stinger was observed on dermoscopy (Figure 1D). After treatment with 0.1% mometasone furoate cream, the lesion completely resolved within 1 day.

Hymenoptera stings are common and painful envenomations that lead to an annual average of 62 deaths in the United States.^1^ The stinging insects of the Hymenoptera order are divided into three groups: Apidae (honeybees, bumblebees), Vespidae (wasps, yellow jackets, hornets), and Formicidae (ants).^2^ Honeybees leave their stingers in the victim’s skin and die shortly after they sting.^3^ Bumblebees have a stinger with fewer barbs and a stronger attachment, enabling them to sting multiple times without dying.^4^ Wasps, yellow jackets, and hornets are also capable of multiple stings, like bumblebees.^4^ Most fire ants sting the lower extremities in clusters.^2^ Unlike bees, wasps, yellow jackets, and hornets, fire ants inject venom slowly, which results in a delayed onset of pain.^3^

Although honeybees are notorious for leaving their stingers in the victim’s skin, other members of the Hymenoptera order may also leave a stinger behind.^4^ Therefore, it is crucial to remove any stinger promptly. The stinger should be extracted by sweeping the dull blade of a butter knife or the edge of a credit card across the skin at an almost parallel angle to the surface.^2^ Using tweezers for removal should be avoided, as it can result in the injection of additional venom into the sting site.^4^ Although dermoscopy is a useful tool to detect stingers left by Hymenoptera stings, only a wasp stinger has been reported in the literature.^5^ To the best of our knowledge, this is the first report of a bee sting observed with dermoscopy. Dermoscopy can be useful for detecting stingers, but its utility in detecting bee stings has not been widely reported.
